# Supplementary figures and images for: Serine-Arginine Protein Kinase 1 Regulates Ebola Virus Transcription
Source: mBio. 2020 Feb 25;11(1):e02565-19. doi: 10.1128/mBio.02565-19 (PMC7042693; doi:10.1128/mBio.02565-19)

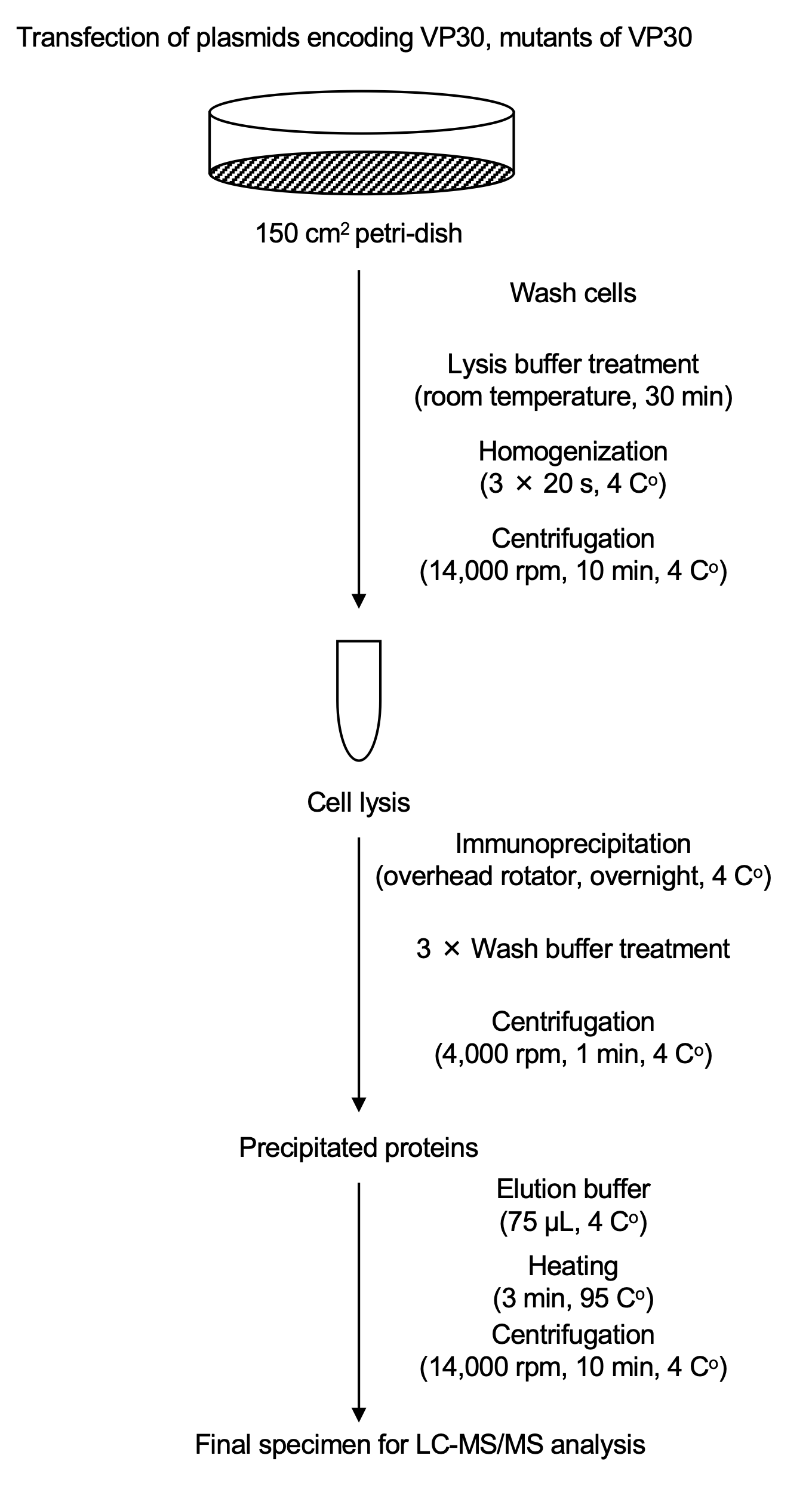

Supplement: FIG S1 [file mBio.02565-19-sf001.tiff]

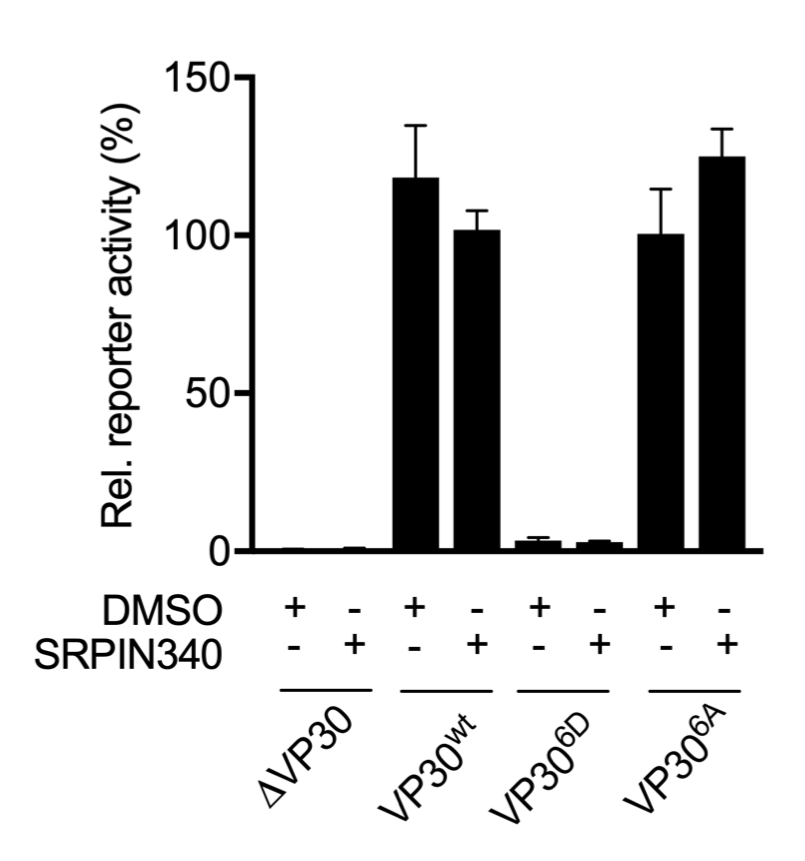

Supplement: FIG S2 [file mBio.02565-19-sf002.tiff]

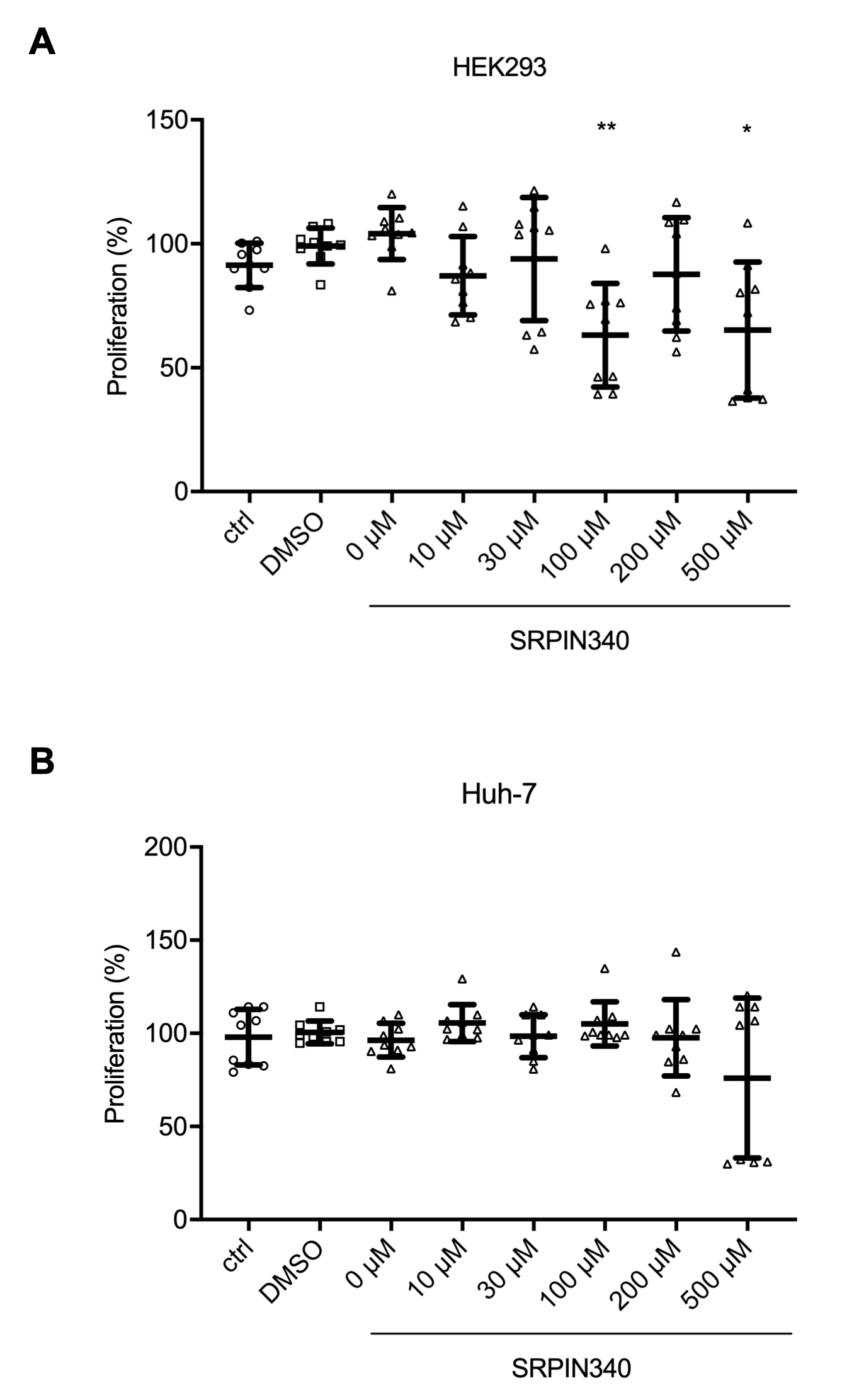

Supplement: FIG S3 [file mBio.02565-19-sf003.tiff]

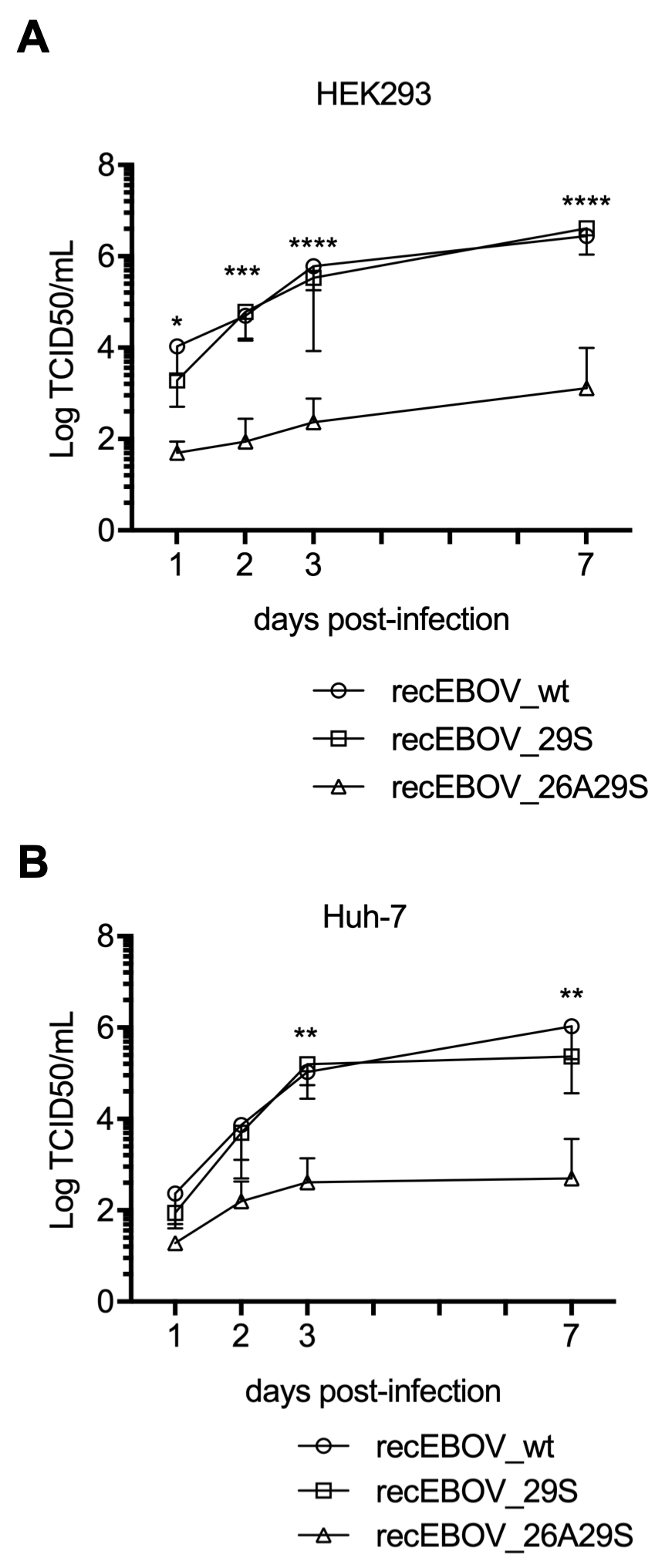

Supplement: FIG S4 [file mBio.02565-19-sf004.tiff]

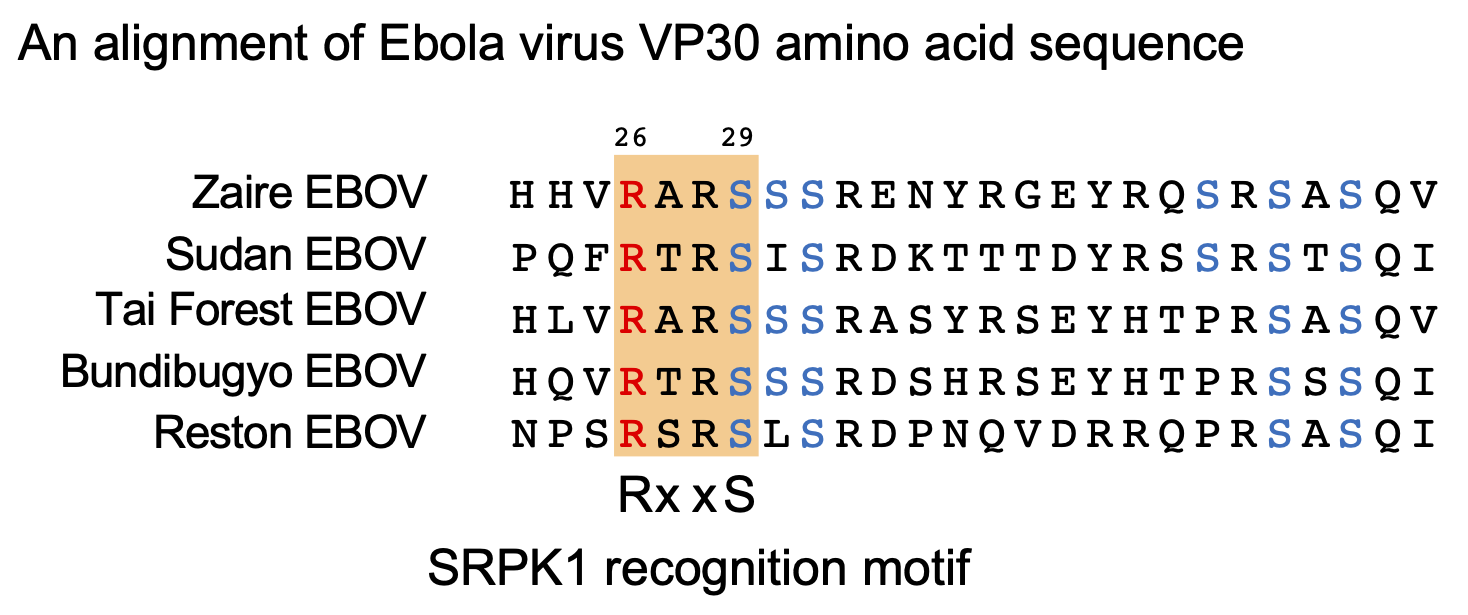

Supplement: FIG S5 [file mBio.02565-19-sf005.tiff]
