# Supplementary material for: Serine-Arginine Protein Kinase 1 Regulates Ebola Virus Transcription
Source: mBio. 2020 Feb 25;11(1):e02565-19. doi: 10.1128/mBio.02565-19 (PMC7042693; doi:10.1128/mBio.02565-19)
Supplement: TABLE S1 [file mBio.02565-19-st001.pdf]

selected results for kinase hits	Proteome Discoverer provided score of indicated VP30 associated kinase											
	Control			VP30^{6A}			VP30^{wt}			VP30^{29S}		
	1st	2nd	3rd	1st	2nd	3rd	1st	2nd	3rd	1st	2nd	3rd
Interferon-induced, double-stranded RNA-activated protein kinase	n.d.	n.d.	n.d.	40.43	n.d.	13.47	83.18	58.93	27.71	79.09	34.23	13.47
Serine-arginine protein kinase 1	n.d.	n.d.	n.d.	70.54	n.d.	41.57	114.80	39.35	39.04	115.69	94.02	44.96
Serine/threonine-protein kinase RIO2	n.d.	n.d.	n.d.	n.d.	n.d.	n.d.	13.18	n.d.	14.07	16.06	n.d.	8.96
Cyclin-dependent kinase 1	n.d.	n.d.	29.52	n.d.	n.d.	n.d.	n.d.	65.14	n.d.	n.d.	30.87	62.67
DNA-dependent protein kinase catalytic subunit	n.d.	212.14	24.18	n.d.	345.79	71.96	n.d.	423.47	39.36	353.24	362.62	80.20
Nucleoside diphosphate kinase B	n.d.	13.00	n.d.	n.d.	27.13	10.30	41.46	8.28	n.d.	n.d.	37.73	8.04
Protein kinase C iota type	n.d.	n.d.	n.d.	23.34	31.48	n.d.	28.67	22.87	n.d.	21.56	20.96	n.d.
Protein kinase C theta type	n.d.	n.d.	n.d.	45.89	40.15	n.d.	n.d.	n.d.	n.d.	51.58	n.d.	n.d.
Pyruvate kinase PKM	9.85	87.78	21.79	91.30	108.79	12.06	87.75	104.25	39.36	67.05	109.24	19.04
Ribose-phosphate pyrophosphokinase 1	18.27	47.01	140.12	n.d.	135.52	n.d.	170.33	140.66	n.d.	n.d.	117.43	n.d.
Ribose-phosphate pyrophosphokinase 2	n.d.	n.d.	125.27	186.31	n.d.	n.d.	153.01	n.d.	n.d.	n.d.	158.34	n.d.
Ribose-phosphate pyrophosphokinase 3	n.d.	n.d.	90.10	146.79	n.d.	n.d.	n.d.	n.d.	n.d.	n.d.	129.60	n.d.
Serine/threonine-protein kinase 38	n.d.	n.d.	71.28	58.23	46.53	n.d.	86.23	n.d.	n.d.	n.d.	74.21	31.88
Serine/threonine-protein kinase N2	n.d.	n.d.	69.07	n.d.	48.27	n.d.	56.14	51.37	n.d.	n.d.	n.d.	53.01
Thymidine kinase, cytosolic	n.d.	n.d.	16.98	n.d.	15.16	n.d.	9.68	24.02	n.d.	n.d.	n.d.	n.d.
Uncharacterized aarF domain-containing protein kinase 2	n.d.	n.d.	n.d.	44.58	61.01	n.d.	n.d.	44.60	n.d.	51.49	38.48	n.d.

n.d.: not detected
